# First case of congenital methemoglobinemia in Nepalese population: a case report

**DOI:** 10.1097/MS9.0000000000001552

**Published:** 2023-11-27

**Authors:** Prashu Ram Bista, Anjila Shrestha, Saloni Shrestha

**Affiliations:** Nepal Medical College and Teaching Hospital, Jorpati, Kathmandu, Nepal

**Keywords:** case report, congenital methemoglobinemia, hereditary, Nepal

## Abstract

**Introduction and importance:**

Congenital methemoglobinemia is a rare hereditary disorder that leads to decreased oxygen delivery to the tissues. The severity of symptoms is directly proportional to the methemoglobin levels in the blood. Furthermore, this is the first case of congenital methemoglobinemia reported in the Nepalese population.

**Case presentation:**

We herein present a case of a 33-year-old male with congenital methemoglobinemia, the first reported case among the Nepalese population. His peripheral oxygen saturation level did not improve despite increasing the oxygen supplementation, and a saturation gap of more than 5% was present. The dark brown color of the blood was noted on the blood sample. On investigations, the methemoglobin level was 9%.

**Clinical discussion:**

Congenital methemoglobinemia can occur due to a deficiency of an enzyme known as cytochrome b5 reductase, which primarily converts methemoglobin to hemoglobin. There are two types of congenital methemoglobinemia, type I and type II which can be distinguished clinically by the presence of neurological impairment and mental retardation, which can be seen in type II congenital methemoglobinemia.

**Conclusion:**

Congenital methemoglobinemia is a rare syndrome and has not been previously reported in the Nepalese population. Although there are various diagnostic clues including relevant medical history, saturation gap of more than 5%, dark brown coloration of blood, and investigations such as methemoglobin level, healthcare services like cytochrome b5 reductase enzymatic activity and molecular genetic testing regarding congenital methemoglobinemia is recommended.

## Introduction

Highlights

- Congenital methemoglobinemia is a rare hereditary disorder that leads to decreased oxygen delivery to the tissues.
- Low oxygen saturation levels despite oxygen supplementation, saturation gaps, and the presence of chocolate brown-colored blood can provide diagnostic clues for congenital methemoglobinemia.
- There are two types of congenital methemoglobinemia, type I and type II which can be distinguished clinically by the presence of neurological impairment and mental retardation, which can be seen in type II congenital methemoglobinemia.

Congenital methemoglobinemia is a rare hereditary disorder that can occur due to a deficiency of an enzyme known as cytochrome b5 reductase (cb5r), which primarily converts methemoglobin to hemoglobin. Congenital methemoglobinemia results in a decrease in oxygen delivery to the tissues, causing hypoxia and cyanosis^1^. The severity of symptoms is directly proportional to the methemoglobin levels in the blood. With increasing severity, patients can present with headaches, fatigue, dizziness, acidosis, arrhythmia, and seizures^2^. There has been no prior case of congenital methemoglobinemia reported in the Nepalese population. Hereby, we report a case of a 33-year-old male diagnosed with congenital methemoglobinemia. This case report has been reported in line with the SCARE 2023 Criteria^3^.

## Timeline

The patient presented to the emergency room of our tertiary care center on 18 August 2023 and was subsequently admitted to the pulmonology ward of the internal medicine department.

## Patient information

A 33-year-old male presented to the emergency department of our tertiary care center with complaints of headache and dizziness for 3 months with an increase in severity for 7 days. Headaches were present over the frontal and temporal regions and were sudden on onset, intermittent, and throbbing in nature. Symptoms appeared after his travel to high altitude. He had no history of altered sensorium, abnormal body movement, and palpitations.

He gave a history of similar episodes of illness in the past. According to the patient, his symptoms resolved on their own and no medical help was sought. His family history revealed a low peripheral oxygen saturation (SpO_2_) level in his brother, and upon further inquiry, his father was also reported to have a low peripheral oxygen saturation level. His two siblings died at a very young age without any known reason. There was no history of exposure to drugs and exposure to any exogenous agents.

### Clinical findings

On examination, the patient was well-oriented to time, place, and person. His body temperature was 98°F (36.66°C), blood pressure 100/60 mmHg, respiratory rate 20 breaths per minute, pulse 92 beats per minute, peripheral oxygen saturation level was 75% under room air and 84% at 2 l/min of supplemental oxygen which did not improve despite increasing the oxygen supplementation. Arterial oxygen saturation (SaO_2_) level was 96.6%. A saturation gap of more than 5% was present. Peripheral oxygen saturation level was 82% after 5 min of exercise. Cardiovascular and respiratory examination showed no significant findings. Neurological examination was unremarkable and there were no signs and symptoms of mental retardation.

### Diagnostic assessment and interpretation

**Figure 1:**
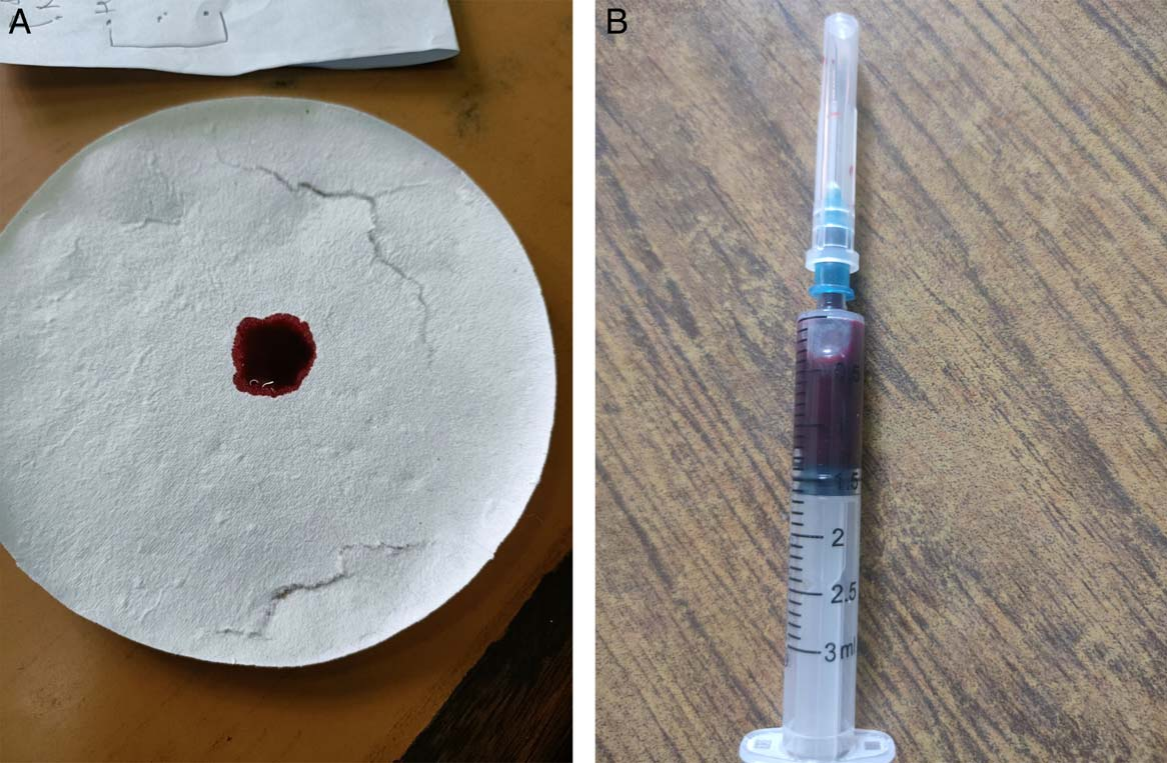
(A) The dark brown color of the blood sample examined in filter paper and (B) the Dark brown color of blood in the syringe.

The dark brown color of the blood was noted on the blood sample (Fig. 1A, B). On investigations, the methemoglobin level was 9%. On arterial blood gas analysis, arterial oxygen saturation (SaO_2_) level was 96.6%, pH was 7.382, pCO_2_ was 45.7 mmHg, and pO_2_ was 81.0 mmHg. On further investigations, no significant findings were reported on hemoglobin electrophoresis and glucose-6-phosphate dehydrogenase (G6PD) enzyme was not deficient, which ruled out a probable diagnosis of hemoglobin M disease and G6PD deficiency, respectively. Investigations to rule out hemolysis and end-organ dysfunction were done. Complete blood count revealed a hemoglobin level of 12 mg/dl, red blood cell count of 4.68 million/μl and leukocyte count of 5450 cells/μl. His bilirubin level was 3.52 mg/dl. Renal function tests revealed urea 20 mg/dl, creatinine 0.82 mg/dl, sodium 142 mmol/l, and potassium 4.1 mmol/l. Peripheral smear showed no abnormal findings. The D-dimer test level was 0.01 mg/l. Chest X-ray, electrocardiogram, and echocardiography was normal. Hence, the conclusion was that features were suggestive of congenital methemoglobinemia; however, investigations like cytochrome b5 reductase enzymatic activity and molecular genetic testing could not be done due to a lack of respective facilities.

### Therapeutic intervention

High-flow oxygen supplementation was given during his stay in the Emergency Room. The patient was then started on oral ascorbic acid tablets 500 mg twice a day for 1 month as he was persistently having headaches and dizziness.

### Follow-up and outcomes

During his first regular follow-up after a month in the outpatient department of internal medicine of our tertiary care center recently, he was symptomatically better and his episodes of headache and dizziness were improving. Oral ascorbic acid tablets 500 mg twice a day were continued twice a day until his next follow-up after a month.

## Discussion

Hemoglobin consists of four heme molecules, each containing an atom of iron. In normal hemoglobin, iron is found in a reduced form known as ferrous (Fe^2+^) state. Fe^2+^ binds with oxygen and helps in delivering oxygen to the tissues. However, if the iron is oxidized into a ferric (Fe^3+^) state, methemoglobin is formed, in which the heme molecule is incapable of transporting oxygen. Cytochrome b5 reductase is an enzyme which converts methemoglobin to hemoglobin. Congenital methemoglobinemia is a rare hereditary condition that can occur either due to a deficiency of cytochrome b5 reductase or hemoglobin variants known as hemoglobin M^1,4^.

Congenital methemoglobinemia results in a decrease in oxygen delivery to the tissues, causing hypoxia and cyanosis^1^. In contrast, cyanosis was not seen in our patient. The severity of symptoms is directly proportional to the methemoglobin levels in the blood. With increasing severity, patients can present with headaches, fatigue, dizziness, acidosis, arrhythmia, and seizures^2^. In our case study, the patient presented with headache and dizziness associated with shortness of breath. Patients with hereditary methemoglobinemia can be completely asymptomatic for many years of their lives^5,6^. Similarly, our patient had a delay in the onset of symptoms. There are two types of congenital methemoglobinemia, type I and type II which can be distinguished clinically by the presence of neurological impairment and mental retardation, which can be seen in type II congenital methemoglobinemia^1^. On examination, the patient did not have any neurological and mental impairment.

Peripheral oxygen saturation level was 84%, which did not improve despite increasing the oxygen supplementation in our case study, which can be seen in patients with congenital methemoglobinemia^7^. The saturation gap is the difference between the low SpO_2_ level and the falsely normal SaO_2_. A saturation gap of more than 5% provides a diagnostic clue for hemoglobinopathy including methemoglobinemia^8^. A saturation gap of more than 5% was present on our study, which provided a hint on the presence of hemoglobinopathy.

Methemoglobinemia results in brown coloration of the blood^9^. Similarly, the dark brown color of the blood was noted on the blood sample. Furthermore, a diagnosis of congenital methemoglobinemia can be made by measuring the methemoglobin concentration, assaying the cb5r enzymatic activity, and conducting molecular genetic testing^1^. Methemoglobin level was 9% in our case study. No significant findings were reported on hemoglobin electrophoresis, which rules out the possibility of congenital methemoglobinemia due to HbM.

Although the resolution of the symptoms of congenital methemoglobinemia when treated with a high dose of ascorbic acid has been reported in the literature, there was no significant improvement in the administration of ascorbic acid in our case study^10^.

### Strength and limitations

This case report is the first reported case among the Nepalese population despite challenges encountered due to a lack of facilities during the workup of congenital methemoglobinemia. Cytochrome b5 reductase enzymatic activity and molecular genetic testing could not be done due to a lack of respective facilities. Hence, we also would like to bring attention to the need for healthcare facilities concerning methemoglobinemia in Nepal.

## Conclusion

Congenital methemoglobinemia is a rare syndrome and has not been previously reported in the Nepalese population. Although there are various diagnostic clues including relevant medical history, saturation gap, dark brown coloration of blood and investigations such as methemoglobin concentrations, healthcare services like cb5r enzymatic activity and molecular genetic testing of the patient as well as his family members are recommended.

## Ethical approval

Case reports are exempt from ethical approval in our institution, Nepal Medical College and Teaching Hospital, Attarkhel, Kathmandu.

## Consent

Written informed consent was obtained from the patient for publication of this case report and accompanying images. A copy of the written consent is available for review by the Editor-in-Chief of this journal on request.

## Source of funding

No source of funding was provided.

## Author contribution

P.R.B., A.S., and S.S.: contributed equally to conceptualization, literature review, manuscript preparation, manuscript editing, and reviewing of the case report. All the authors individually did the final proofreading of the manuscript before submission.

## Conflicts of interest disclosure

The authors have no conflicts of interest.

## Research registration unique identifying number (UIN)

The case report does not have registration.

## Guarantor

Dr Prashu Ram Bista, Nepal Medical College and Teaching Hospital, Jorpati, Kathmandu, Nepal; e-mail: prashuramaiims@gmail.com, Tel.: +977 9841947977.

## Data availability statement

Yes. The details and investigations of the case in the study will be available on request.

## Provenance and peer review

Not commissioned, externally peer-reviewed.
